# 

**Published:** 1953

**Authors:** 


### THE MEDICAL JOURNAL OF THE SOUTH-WEST

*Incorporating*
*The Bristol Medico-Chirurgical Journal*

W1 ME359

ARMED FORCES

FEB 3 1954

MEDICAL LIBRARY

1953

